# Postoperative *Staphylococcus aureus* Infections in Patients With and Without Preoperative Colonization

**DOI:** 10.1001/jamanetworkopen.2023.39793

**Published:** 2023-10-31

**Authors:** Darren P. R. Troeman, Derek Hazard, Leen Timbermont, Surbhi Malhotra-Kumar, Cornelis H. van Werkhoven, Martin Wolkewitz, Alexey Ruzin, Herman Goossens, Marc J. M. Bonten, Stephan Harbarth, Frangiscos Sifakis, Jan A. J. W. Kluytmans, and the ASPIRE-SSI Study Team, Jelle Vlaeminck, Tuba Vilken, Basil Britto Xavier, Christine Lammens, Marjolein van Esschoten, Fleur P. Paling, Claudia Recanatini, Frank Coenjaerts, Bret Sellman, Christine Tkaczyk, Susanne Weber, Miquel Bart Ekkelenkamp, Lijckle van der Laan, Bastiaan P. Vierhout, Elodie Couvé-Deacon, Miruna David, David Chadwick, Martin J. Llewelyn, Andrew Ustianowski, Antony Bateman, Damian Mawer, Biljana Carevic, Sonja Konstantinovic, Zorana Djordjevic, María Dolores del Toro-López, Juan Pablo Horcajada Gallego, Dolores Escudero, Miquel Pujol Rojo, Julian Torre-Cisneros, Francesco Castelli, Giuseppe Nardi, Pamela Barbadoro, Mait Altmets, Piret Mitt, Adrian Todor, Serban-Ion Bubenek-Turconi, Dan Corneci, Dorel Săndesc, Valeriu Gheorghita, Radim Brat, Ivo Hanke, Jan Neumann, Tomáš Tomáš, Wim Laffut, Anne-Marie Van den Abeele

**Affiliations:** 1 Julius Center for Health Sciences and Primary Care, University Medical Center Utrecht, Utrecht University, Utrecht, the Netherlands; 2 Institute of Medical Biometry and Statistics, Faculty of Medicine and Medical Center, University of Freiburg, Freiburg, Germany; 3 Laboratory of Medical Microbiology, Vaccine and Infectious Disease Institute, University of Antwerp, Antwerp, Belgium; 4 Microbial Sciences, R&D BioPharmaceuticals, AstraZeneca Plc, Gaithersburg, Maryland; 5 Infection Control Programme and World Health Organization Collaborating Center, Geneva University Hospitals and Faculty of Medicine, Geneva, Switzerland; 6 now with Gilead Sciences Inc, Foster City, California; 7 AstraZeneca Plc, Gaithersburg, Maryland; 8 Department of Medical Microbiology, University Medical Center Utrecht, Utrecht University, Utrecht, the Netherlands; 9 University Medical Center Utrecht, Utrecht University, Utrecht, the Netherlands; 10 Department of Surgery, Amphia Hospital, Breda, North Brabant, the Netherlands; 11 Department of Surgery, Wilhelmina Ziekenhuis Assen, Assen, Drenthe, the Netherlands; 12 Centre Hospitalier Universitaire de Limoges, Limoges, France; 13 Queen Elizabeth Hospital Birmingham, University Hospitals Birmingham National Health Service (NHS) Foundation Trust, Birmingham, England, United Kingdom; 14 South Tees Hospitals NHS Foundation Trust, Middlesbrough, England, United Kingdom; 15 University Hospitals Sussex NHS Foundation Trust, Brighton, United Kingdom; 16 North Manchester General Hospital, Pennine Acute Hospitals NHS Trust, Manchester, England, United Kingdom; 17 University Hospitals of Derby & Burton NHS Foundation Trust, Derby, England, United Kingdom; 18 York and Scarborough Teaching Hospitals NHS Foundation Trust, York, England, United Kingdom; 19 Clinical Centre of Serbia, Belgrade, Serbia; 20 Institute for Orthopedic Surgery Banjica, Belgrade, Serbia; 21 Clinical Centre of Kragujevac, Kragujevac, Serbia; 22 Hospital Universitario Virgen Macarena, Seville, Spain; 23 Hospital del Mar-IMIM, UPF, Barcelona, Spain; 24 CIBERINFEC, Instituto de Salud Carlos III, Madrid, Spain; 25 Hospital Universitario de Asturias, Asturia, Spain; 26 Hospital Universitario de Bellvitge, Barcelona, Spain; 27 Hospital Universitario Reina Sofía-IMIBIC, Cordoba, Spain; 28 Hospital Brescia, University of Brescia, Brescia, Italy; 29 UOC Anestesia e Rianimazione, Ospedale Infermi, Rimini, Italy; 30 Azienda Ospedaliera Universitaria Ospedali Riuniti, Ancona, Italy; 31 North Estonia Medical Centre, Tallinn, Estonia; 32 Tartu University Hospital, Tartu, Estonia; 33 Emergency County Hospital Cluj Napoca, Cluj-Napoca, Romania; 34 Prof. Dr C.C. Iliescu Institute for Emergency Cardiovascular Diseases, Bucharest, Romania; 35 Elias Emergency University Hospital, Bucharest, Romania; 36 Timisoara County Hospital, Timisoara, Romania; 37 Central Military University Emergency Hospital “Dr Carol Davila,” Bucharest, Romania; 38 University Hospital Ostrava, Ostrava-Poruba, Czechia; 39 University Hospital Hradec Králové, Hradec Králové, Czechia; 40 Motol University Hospital, Prague, Czechia; 41 St. Anne's University Hospital, Brno, Czechia; 42 Heilig Hart Hospital, Lier, Belgium; 43 AZ Sint-Lucas Ziekenhuis Gent-Campus Volkskliniek, Gent, Belgium

## Abstract

**Importance:**

*Staphylococcus aureus* surgical site infections (SSIs) and bloodstream infections (BSIs) are important complications of surgical procedures for which prevention remains suboptimal. Contemporary data on the incidence of and etiologic factors for these infections are needed to support the development of improved preventive strategies.

**Objectives:**

To assess the occurrence of postoperative *S aureus* SSIs and BSIs and quantify its association with patient-related and contextual factors.

**Design, Setting, and Participants:**

This multicenter cohort study assessed surgical patients at 33 hospitals in 10 European countries who were recruited between December 16, 2016, and September 30, 2019 (follow-up through December 30, 2019). Enrolled patients were actively followed up for up to 90 days after surgery to assess the occurrence of *S aureus* SSIs and BSIs. Data analysis was performed between November 20, 2020, and April 21, 2022. All patients were 18 years or older and had undergone 11 different types of surgical procedures. They were screened for *S aureus* colonization in the nose, throat, and perineum within 30 days before surgery (source population). Both *S aureus* carriers and noncarriers were subsequently enrolled in a 2:1 ratio.

**Exposure:**

Preoperative *S aureus* colonization.

**Main Outcomes and Measures:**

The main outcome was cumulative incidence of *S aureus* SSIs and BSIs estimated for the source population, using weighted incidence calculation. The independent association of candidate variables was estimated using multivariable Cox proportional hazards regression models.

**Results:**

In total, 5004 patients (median [IQR] age, 66 [56-72] years; 2510 [50.2%] female) were enrolled in the study cohort; 3369 (67.3%) were *S aureus* carriers. One hundred patients developed *S aureus* SSIs or BSIs within 90 days after surgery. The weighted cumulative incidence of *S aureus* SSIs or BSIs was 2.55% (95% CI, 2.05%-3.12%) for carriers and 0.52% (95% CI, 0.22%-0.91%) for noncarriers. Preoperative *S aureus* colonization (adjusted hazard ratio [AHR], 4.38; 95% CI, 2.19-8.76), having nonremovable implants (AHR, 2.00; 95% CI, 1.15-3.49), undergoing mastectomy (AHR, 5.13; 95% CI, 1.87-14.08) or neurosurgery (AHR, 2.47; 95% CI, 1.09-5.61) (compared with orthopedic surgery), and body mass index (AHR, 1.05; 95% CI, 1.01-1.08 per unit increase) were independently associated with *S aureus* SSIs and BSIs.

**Conclusions and Relevance:**

In this cohort study of surgical patients, *S aureus* carriage was associated with an increased risk of developing *S aureus* SSIs and BSIs. Both modifiable and nonmodifiable etiologic factors were associated with this risk and should be addressed in those at increased *S aureus* SSI and BSI risk.

## Key Points

**Question:**

What is the cumulative incidence of *Staphylococcus aureus* surgical site infections (SSIs) and bloodstream infections (BSIs) in Europe, and what factors are associated with an increased risk of SSIs and BSIs?

**Findings:**

In a cohort study of 5004 surgical patients, the weighted cumulative incidence of *S aureus* SSIs and BSIs was 1.23%. Preoperative *S aureus* carriage, mastectomy or neurosurgery, higher body mass index, and having nonremovable implants in the body were independently associated with *S aureus* SSIs and BSIs.

**Meaning:**

*Staphylococcus aureus* SSIs and BSIs are important postoperative complications, and future interventions aimed at prevention of these infections should focus on at-risk surgical patient groups to achieve a higher efficacy.

## Teaser

This cohort study assesses the incidence of *Staphylococcus aureus* surgical site and bloodstream infections and examines factors associated with these infections among surgical patients with and without preoperative *S aureus* colonization.

## Introduction

Surgical site infections (SSIs) and postoperative bloodstream infections (BSIs) are important and common complications of surgical procedures,^1,2^ causing significant morbidity, extended hospital stays, and increased mortality and health care costs.^3,4^ A considerable proportion of these postoperative infections are caused by *Staphylococcus aureus*.^5,6,7^ In recent years, the management of postoperative *S aureus* infections has been complicated by the increase and spread of antibiotic-resistant *S aureus*.^8^ Because postoperative *S aureus* infections can have severe consequences for patients, there is an increasing need for effective interventions aimed at prevention and management of these infections. To support the development of these interventions, contemporary information about the incidence of and etiologic factors associated with *S aureus* postoperative infections is needed. The epidemiology of *S aureus* SSIs in Europe has not been fully described in part because of variations in case definitions and surveillance systems and a lack of comprehensive preoperative *S aureus* screening programs. Therefore, we attempted to assess the incidence and quantify the association of patient-related and contextual factors with *S aureus* SSIs and postoperative BSIs in Europe.

## Methods

### Study Design and Setting

ASPIRE-SSI (Advanced Understanding of *Staphylococcus aureus* Infections in Europe–SSI)^9^ was a prospective multicenter cohort study that recruited surgical patients at 33 hospitals in 10 European countries between December 16, 2016, and September 30, 2019. The last study patient completed follow-up on December 30, 2019. In each European subregion, as described by the United Nations,^10^ at least 2 countries were included. The study design and rationale have been described in detail elsewhere.^11^ Additional methods are reported in the eMethods in Supplement 1. Institutional or ethics review boards at each participating hospital or country approved the study protocol. All participants provided written informed consent. This study was conducted in accordance with the principles of the Declaration of Helsinki,^12^ the Medical Research Involving Human Subjects Act,^13^ and local guidelines in the participating countries. This study followed the Strengthening the Reporting of Observational Studies in Epidemiology (STROBE) reporting guideline.^14^

### Study Participants

Patients 18 years or older undergoing 11 different types of surgical procedures were screened for *S aureus* colonization in the nose, throat, and perineum within 30 days before surgery and provided a preoperative serum sample. These patients constituted the source population. Subsequent enrollment in the study cohort (main study population) was determined by the preoperative *S aureus* colonization status of the patients, completeness of preoperative sample collection, and whether the surgery took place. Within each surgical procedure, all preoperative *S aureus* carriers were eligible for enrollment in the study cohort, together with the first eligible noncarrier after 2 enrolled *S aureus* carriers. The aim was to enroll 5000 study cohort participants. Exclusion criteria were simultaneous participation in any antistaphylococcal intervention study, an SSI as the reason for undergoing surgery, and expected inability to comply with study procedures or follow-up. The rationale for the sample size is described elsewhere.^11^ Postoperative follow-up data were collected for all patients at days 7, 14, 21, 28, 60, and 90 after surgery (±3 days each) by medical record review and by contacting the participants or next of kin. If a postoperative infection was suspected, the participant was encouraged to seek medical attention for clinical assessment and collection of microbiological cultures. The list of participating countries and the number of enrolled participants per country are given in eTable 1 in Supplement 1.

### Data Collection and Outcomes

Clinical reasoning and a literature review guided the selection of variables that were collected in this study. A description of the collected variables is given in the eMethods in Supplement 1. All data were recorded in web-based case report forms. For this study, *S aureus* SSIs and postoperative BSIs were combined into 1 composite outcome. *Staphylococcus aureus* SSIs and postoperative BSIs were defined as the isolation of *S aureus* from a wound-related (eg, culture from surgical site or purulent drainage) or blood culture, respectively, and fulfilling the criteria for an SSI^15^ or BSI (criterion laboratory-confirmed bloodstream infection 1)^16^ according to the Centers for Disease Control and Prevention guidelines.

### Laboratory Methods

*Staphylococcus aureus* screening samples were analyzed locally on chromogenic culture media (Colorex staph aureus; BioTrading Benelux B.V.) using standardized methods. The presence of *S aureus* was based on phenotypic criteria (pink- or mauve-colored colonies). This culture medium has a reported sensitivity of 95.5% and a specificity of 99.4% for the detection of *S aureus*.^17^ All collected *S aureus* strains, including from clinical samples in the case of SSIs or BSIs, were frozen at −80 °C and shipped to the central laboratory for further analysis. All received isolates (n = 4738; 3345 participants) were aerobically cultured on blood agar (defibrinated horse blood; E & O Laboratories Ltd) at 37 °C for 18 to 24 hours and identified using matrix-assisted laser desorption/ionization–time-of-flight mass spectrometry (Bruker Corp). Methicillin susceptibility of all confirmed *S aureus* strains (n = 4427; 3148 participants) was determined using high-throughput screening for cefoxitin resistance. Briefly, bacterial suspensions of 0.5 McFarland were spotted on methicillin-resistant *S aureus* (MRSA) chromogenic media (CHROMagar; BioTrading) using a multiblot replicator (VP 407-96 Pin Multi-Blot Replicator [9-mm centers, 2.36-mm pin diameter, 22-mm long, blunt tip]; V&P Scientific Inc), followed by aerobic incubation at 37 °C for 18 to 24 hours. Suspected MRSA was confirmed by a cefoxitin E-test (bioMérieux). Cefoxitin minimum inhibitory concentration greater than 4 μg/mL was indicative of MRSA according to the European Committee on Antimicrobial Susceptibility Testing breakthrough guidelines, version 10.0. Additionally, all available *S aureus* isolates from the patients who developed *S aureus* SSIs and BSIs (screening isolates [n = 84; 54 patients] and infecting isolates [n = 139; 60 patients]) and from a random sample of noninfected patients (n = 221; 162 patients) were characterized by multilocus sequence typing using whole-genome sequencing data (eFigure 1 in Supplement 1). Genomic DNA was extracted from *S aureus* isolates using a high-molecular-weight DNA extractor (MagAttract HMW DNA Kit; Qiagen), per the manufacturer’s instructions, and quantified with a double-stranded DNA high-sensitivity assay (Qubit dsDNA HS assay kit; Thermo Fisher Scientific). Libraries were then generated (NexteraXT DNA sample preparation kit; Illumina Inc) and sequenced (2 × 250 bp) with a sequencer (MiSeq; Illumina Inc). FastQC, version 0.11.7 (Babraham Bioinformatics) was used to assess the quality of the raw sequence data. Trimming of reads, building a draft genome, and multilocus sequence typing were performed using BacPipe, version 1.2.6.^18^

### Statistical Analysis

Baseline characteristics are presented in aggregate and stratified by preoperative *S aureus* colonization status. Study estimates were determined in the study cohort and estimated for the source population using weighting methods.^19^ These weighting methods considered the likelihood of patients to be enrolled in the study cohort to obtain study estimates for the underlying source population. A detailed description of these methods is given in the eMethods in Supplement 1.

#### Incidence Calculation

The 90-day cumulative incidence of *S aureus* SSIs and BSIs (only the first episode per patient) was calculated for the entire weighted study population and for *S aureus* carriers and noncarriers separately. The 95% CIs were determined by bootstrapping and applying the weights.

#### Risk Factor Analysis

Univariable cause-specific Cox proportional hazards regression models were used to assess associations between each explanatory variable and (1) the outcome *S aureus* SSIs and BSIs (with censoring for loss to follow-up and death) and (2) the competing event death without *S aureus* SSIs and BSIs (with censoring for loss to follow-up and *S aureus* SSIs and BSIs). The latter was done to assess whether any variable could also be associated with informative censoring by being associated with the competing events and thus affect the hazard ratio (HR) of explanatory variables for *S aureus* SSIs and BSIs. This process yielded univariable cause-specific HRs. Because of anticipated differences among countries, we defined country as a stratum variable. Variables with a *P* ≤ .157 (corresponding to selection via Akaike information criterion optimization) in either analysis were included in a multivariable model to quantify the relative rates of developing *S aureus* SSIs and BSIs. A 2-sided *P* < .05 was considered significant. All analyses were conducted on weighted and unweighted data.

#### Missing Data

We assumed data were missing at random and used default multivariate imputation by chained equation procedures to acquire 5 suitable imputed data sets for the main analyses. Model estimates were pooled according to Rubin’s rules^20^ to provide a single mean estimate and adjusted SEs for each variable. Using these results, we derived pooled (adjusted) HRs and robust 95% CIs for each variable.

#### Sensitivity Analysis

We conducted several sensitivity analyses to assess the robustness of the study results. First, we repeated the multivariable analysis while keeping preoperative *S aureus* decolonization in the model because it is an important confounder of the occurrence association between preoperative *S aureus* colonization and postoperative *S aureus* infection. Second, we repeated the univariable and multivariable analyses using the subdistribution hazards (Fine and Gray)^21^ approach to assess the independent associations between the explanatory variables and the risk of developing *S aureus* SSIs and BSIs. Third, we conducted a complete case analysis to assess whether the imputation procedure had any effect on the study results. All statistical analyses were performed between November 20, 2020, and April 21, 2022, using R statistical software, version 4.0.2 (R Foundation for Statistical Computing).^22^

## Results

In total, 5004 patients (median [IQR] age, 66 [56-72] years; 2510 [50.2%] female and 2494 [49.8%] male) were included in the study cohort; 3369 patients (67.3%) were *S aureus* carriers. Table 1 gives the characteristics of the *S aureus* carriers and noncarriers of the study cohort. Compared with the noncarriers, carriers were more often male, had a prior history of *S aureus* colonization or infection, and received preoperative topical decolonization treatment. Other characteristics were similar. The characteristics of the weighted and original source population are presented in eTable 2 in Supplement 1.

**Table 1.** Baseline Characteristics of Study Participants^a^

Characteristic	Unweighted population	
	*Staphylococcus aureus* carriers (n = 3369)	Noncarriers (n = 1635)
Sex		
Female	1642 (48.7)	868 (53.1)
Male	1727 (51.3)	767 (46.9)
Implant before surgery		
No	2544 (75.5)	1246 (76.2)
Yes	820 (24.3)	387 (23.7)
Missing	5 (0.1)	2 (0.1)
Type of surgery		
Cardiovascular surgery^b^	1002 (29.7)	488 (29.8)
Orthopedic surgery^c^	1231 (36.5)	609 (37.2)
Neurosurgery^d^	612 (18.2)	287 (17.6)
Emergency surgery	207 (6.1)	99 (6.1)
Mastectomy	317 (9.4)	152 (9.3)
ASA score		
1	326 (9.7)	136 (8.3)
2	1445 (42.9)	707 (43.2)
3	1269 (37.7)	640 (39.1)
≥4	187 (5.6)	93 (5.7)
Missing	142 (4.2)	59 (3.6)
Receipt of immunosuppressive medication within 2 weeks of surgery		
No	3194 (94.8)	1559 (95.4)
Yes	171 (5.1)	75 (4.6)
Missing	4 (0.1)	1 (0.1)
History of *S aureus* colonization or infection		
No	3033 (90)	1589 (97.2)
Yes	329 (9.8)	43 (2.6)
Missing	7 (0.2)	3 (0.2)
Receipt of preoperative decolonization treatment		
No	2538 (75.3)	1353 (82.8)
Yes	831 (24.7)	282 (17.2)
Age, median (IQR), y	65 (55;72)	67 (58;73)
Missing	0	0
BMI, median (IQR)	27.9 (24.9-31.6)	27.7 (24.8-31.1)
Missing	44 (1.3)	23 (1.4)
CCI, median (IQR)	1 (0-2)	1 (0-2)
Missing	1 (0.0)	2 (0.1)

Abbreviations: ASA, American Society of Anesthesiologists; BMI, body mass index (calculated as weight in kilograms divided by height in meters squared); CCI, Charlson Comorbidity Index.

^a^ Data are presented as number (percentage) unless otherwise indicated.

^b^ Including open cardiac surgery, implantable cardioverter defibrillator implantation, peripheral artery bypass surgery, and central artery reconstructive surgery.

^c^ Including hip prosthesis and knee prosthesis surgery.

^d^ Including craniotomy, laminectomy, and spinal fusion surgery.

### Patient Recruitment

**Figure 1.**
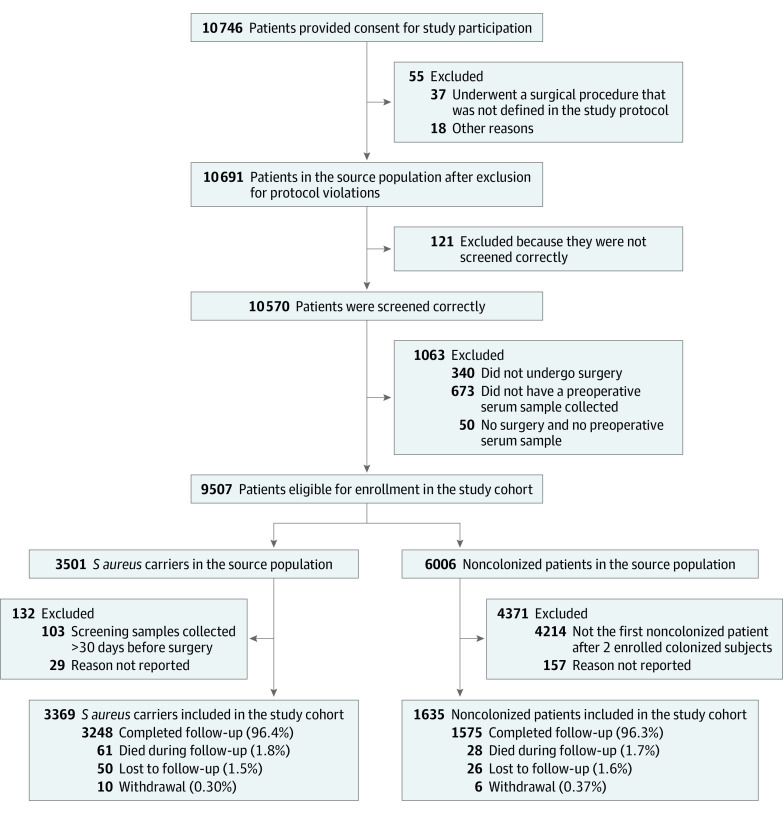
Flow of Participants in the Advanced Understanding of *Staphylococcus aureus* Infections in Europe–Surgical Site Infections (ASPIRE-SSI) Study

In all, 10 691 patients were included in the source population; 121 patients were not screened correctly, and 1063 patients did not undergo surgery or missed serum sample collection. Of the remaining 9507 patients, 3501 (36.8%) were *S aureus* carriers and 6006 (63.2%) noncarriers. For the current analysis, 3369 (67.3%) *S aureus* carriers and 1635 (32.7%) noncarriers were included in the study cohort. Figure 1 shows the flow of patient recruitment and reasons for nonparticipation at each stage.

### Incidence of *S aureus* SSIs and BSIs

A total of 100 study cohort participants developed a *S aureus* SSI or BSI during follow-up (91 SSIs, 4 BSIs, and 5 SSIs with secondary BSIs). Eighty-six (86.0%) of these patients were preoperative *S aureus* carriers. Of the 96 *S aureus* SSIs, 47 (49.0%) were superficial, 34 (35.4%) were deep, and 15 (15.6%) were organ-space SSIs.

**Figure 2.**
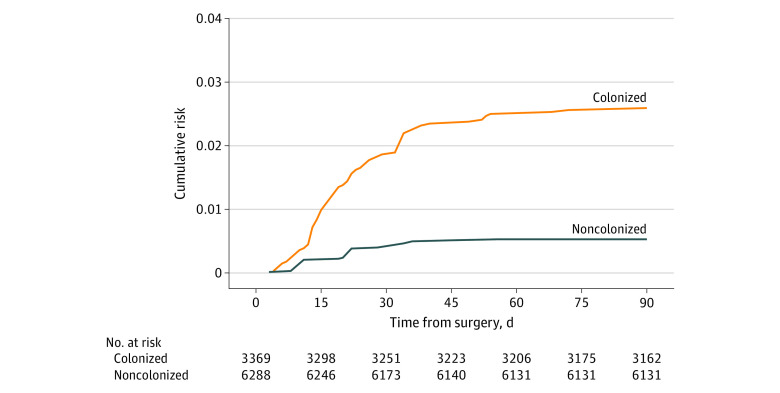
Cumulative Incidence Function for *Staphylococcus aureus* Surgical Site Infections (SSIs) and Bloodstream Infections (BSIs) in *S aureus* Colonized vs Noncolonized Patients

Within the weighted study cohort (n = 9657), 119 participants developed an *S aureus* SSI or BSI. Eighty-six participants were *S aureus* carriers and 33 were noncarriers. The weighted cumulative incidence of *S aureus* SSIs and BSIs was 1.23% (95% CI, 0.97%-1.58%) for the entire weighted study population, 2.55% (95% CI, 2.05%-3.12%) for *S aureus* carriers, and 0.52% (95% CI, 0.22%-0.91%) for noncarriers (Table 2 and Figure 2). There were also differences in the incidence of *S aureus* SSIs and BSIs among different surgical procedures (eTable 3 in Supplement 1). Unweighted incidence calculations are provided in eTable 4 and eFigure 2 in Supplement 1.

**Table 2.** Weighted Cumulative Incidences by Preoperative *Staphylococcus aureus* Colonization Status^a^

*S aureus* colonization status	No. of patients	No. of *S aureus* SSI and BSI events	Cumulative incidence per 100 patients (95% CI)	Time to event, median (IQR), d
Carrier	3369	86	2.55 (2.05-3.12)	19 (13-33)
Noncarrier	6288	33	0.52 (0.22-0.91)	22 (11-28)
Overall	9657	119	1.23 (0.97-1.58)	20 (13-33)

Abbreviations: BSI, bloodstream infection; SSI, surgical site infection.

^a^ The numbers of patients and events given in the table are the weighted totals. These numbers were derived by bootstrapping. A total of 10 000 bootstrap samples of the study cohort were made, after which the bootstrap samples were inflated using the weights (creating 10 000 weighted bootstrap samples). In each weighted bootstrap sample, the cumulative incidence of *S aureus* SSIs and BSIs was calculated for *S aureus* carriers and noncarriers. The sequence of 10 000 cumulative incidences for *S aureus* carriers and noncarriers was used to derive the median cumulative incidence with 95% CIs.

### Prevalence of MRSA Carriage

Of the 4230 screening *S aureus* isolates analyzed (3138 participants), 162 (127 participants) were MRSA. Thus, MRSA carriage was observed in 127 of 3138 *S aureus* carriers (4.0%); the remaining isolates were methicillin-susceptible *S aureus* (eTable 5 in Supplement 1).

### High Concordance in Sequence Type Between Colonizing and Infecting *S aureus*

Among patients who developed an *S aureus* SSI or BSI, the colonizing and infecting *S aureus* isolates were distributed into 23 and 25 sequence types (STs), respectively. The colonizing *S aureus* isolated from the randomly selected cohort of noninfected patients was distributed into 37 STs. The most prevalent STs in patients who developed an *S aureus* SSI or BSI and in the randomly selected cohort participants were STs 30, 45, 5, 8, and 398, with no apparent preference of certain STs to cause infection (eTable 6 in Supplement 1). For 53 patients who developed an *S aureus* infection, both colonizing and infecting *S aureus* strains were available. For 44 of these 53 patients (83.0%), the ST of the colonizing and infecting strains was identical.

### Risk Factor Analysis

A total of 364 records (7.3%) included missing values. Primary reasons for missing data were that the data could not be retrieved from the medical records or that the perineum screening sample was not collected. Results from the weighted univariable and multivariable analyses are presented in Table 3.

**Table 3.** Weighted Risk Factor Analysis for *Staphylococcus aureus* SSIs and BSIs

Risk factor	Univariable analysis for the association with *S aureus* SSIs and BSIs		Univariable analysis for the association with death without *S aureus* SSIs and BSIs		Multivariable analysis for the association with *S aureus* SSIs and BSIs^a^	
	Crude HR (95% CI)	*P* value	Crude HR (95% CI)	*P* value	Adjusted HR (95% CI)	*P* value
Preoperative *S aureus* colonization status^b^						
Colonized	4.56 (2.26-9.21)	<.001	1.17 (0.70-1.97)	.55	4.38 (2.19-8.76)	<.001
Noncolonized	1 [Reference]		1 [Reference]		1 [Reference]	
Sex						
Male	1.35 (0.79-2.30)	.27	1.41 (0.83-2.39)	.20	Not included	Not applicable
Female	1 [Reference]		1 [Reference]			
Nonremovable implant before surgery	1.56 (0.91-2.67)	.10	0.48 (0.24-0.99)	.05	2.00 (1.15-3.49)	.01
Type of surgery						
Cardiovascular surgery	2.13 (1.10-4.12)	.03	5.55 (2.34-13.21)	<.001	1.91 (0.86-4.22)	.11
Mastectomy	4.12 (1.52-11.20)	006	0.44 (0.06-3.44)	.43	5.13 (1.87-14.08)	.002
Neurosurgery	2.37 (1.01-5.55)	.05	12.80 (5.23-31.33)	<.001	2.47 (1.09-5.61)	.03
Emergency surgery	1.78 (0.54-5.84)	.35	7.40 (2.37-23.06)	.001	2.42 (0.67-8.84)	.18
Orthopedic surgery	1 [Reference]	Not applicable	1 [Reference]	Not applicable	1 [Reference]	Not applicable
Having no postoperative drains	0.82 (0.47-1.41)	.47	1.52 (0.80-2.88)	.21	Not included	Not applicable
ASA score						
1	0.73 (0.32-1.71)	.47	0.63 (0.13-3.06)	.57	0.59 (0.26-1.34)	.21
3	1.46 (0.80-2.69)	.22	3.30 (1.48-7.39)	.004	1.49 (0.63-3.55)	.37
≥4	1.66 (0.67-4.12)	.28	7.91 (3.17-19.76)	<.001	1.70 (0.59-4.87)	.33
2	1 [Reference]	Not applicable	1 [Reference]	Not applicable	Not included	Not applicable
Immunosuppressive medication within 2 weeks before surgery	1.77 (0.72-4.36)	.21	3.88 (2.29- 6.55)	<.001	1.74 (0.62-4.91)	.30
Prior history of *S aureus* colonization or infection	1.11 (0.46-2.67)	.82	2.79 (0.47-16.50)	.26	Not included	Not applicable
Preoperative decolonization	1.35 (0.75-2.44)	.32	1.09 (0.49- 2.47)	.83	Not included	Not applicable
Age^c^	0.99 (0.97-1.00)	.03	1.03 (0.99- 1.06)	.12	0.98 (0.97-1.00)	.08
BMI^d^	1.04 (1.01-1.08)	.009	0.97 (0.90-1.05)	.48	1.05 (1.01-1.08)	.008
CCI^e^	1.16 (1.05-1.28)	.003	1.49 (1.36- 1.64)	<.001	1.09 (0.97-1.22)	.17

Abbreviations: ASA, American Society of Anesthesiologists; BMI, body mass index (calculated as weight in kilograms divided by height in meters squared); BSI, bloodstream infection; CCI, Charlson Comorbidity Index; HR, hazard ratio; SSI, surgical site infection.

^a^ Risk factors with a *P* ≤ .157 in either univariable analysis were selected for the multivariable analysis.

^b^ Colonization status before surgery based on *S aureus* screening of the nose, throat, and perineum.

^c^ Per 1-year increase in age (range, 18-99 years).

^d^ Per 1-point increase in BMI (range, 13.5-65.8).

^e^ Per 1-point increase in the CCI (range, 0-12).

The adjusted HR (AHR) for *S aureus* carriers compared with noncarriers for developing *S aureus* SSIs and BSIs was 4.38 (95% CI, 2.19-8.76). Having any noninfected and nonremovable body implant preoperatively (AHR, 2.00; 95% CI, 1.15-3.49), undergoing mastectomy (AHR, 5.13; 95% CI, 1.87-14.08) or neurosurgery (AHR, 2.47; 95% CI, 1.09-5.61) (compared with orthopedic surgery), and having an elevated body mass index (BMI) (AHR, 1.05; 95% CI, 1.01-1.08 per unit increase) also increased the daily likelihood of *S aureus* SSIs and BSIs. The unweighted AHRs are presented in eTable 7 in Supplement 1.

### Sensitivity Analyses

The association of each explanatory variable with the outcome *S aureus* SSIs and BSIs were reassessed in three sensitivity analyses. Repeating the main analyses while keeping preoperative *S aureus* decolonization in the multivariable analysis (eTable 8 in Supplement 1), using the subdistribution hazards approach (eTable 9 in Supplement 1), or using complete data only (eTable 10 in Supplement 1) did not yield any relevant changes in the reported estimates or conclusions.

## Discussion

In this large, prospective cohort study, we found that preoperative *S aureus* carriage is still a major etiologic factor for *S aureus* SSIs and BSIs. The finding that in 83% of colonized patients who developed an *S aureus* SSI or BSI the colonizing and infecting *S aureus* strain had identical STs further supports this association. Additionally, we found that certain types of surgery (mastectomy and neurosurgery), an increasing BMI, and having nonremovable implants also independently increased the risk of *S aureus* SSIs and BSIs. Interestingly, preoperative colonization and postoperative infection with MRSA remained uncommon.

Our study confirms findings from previous studies^23,24,25^ that endogenous *S aureus* carriage is an important etiologic factor for postoperative *S aureus* infections. However, external factors probably also play a significant role in the etiology of postoperative *S aureus* infections. Furthermore, we observed a low level of preoperative MRSA carriage, whereas a higher MRSA prevalence has been reported in several countries.^26,27^ This finding could be attributable to a decreasing trend in MRSA prevalence^8^ or to the finding that the previously reported high prevalence of MRSA carriage was primarily health care associated and not community based.

An interesting finding was that *S aureus* ST398 was found in isolates from study patients (mostly from Northern and Southern Europe) and that it was identified in approximately 15% of *S aureus* SSI and BSI events. This ST was originally discovered in livestock animals in the early 2000s.^28^ Since then, there have been multiple reports of this ST causing infections in humans, especially among those working near livestock or other farm animals, although ST398 *S aureus* strains (predominantly methicillin susceptible) also cause infections in the absence of livestock exposure.^29^ Our data suggest that this *S aureus* ST has become a more common colonizing and infecting strain in certain parts of Europe, although it did not seem to cause more SSIs and BSIs than other STs in our study population.

A high BMI is a well-established risk factor for noncommunicable diseases, such as cardiovascular disease or type 2 diabetes.^30^ However, its role in postoperative outcomes remains a topic of debate, with some studies suggesting that patients with a higher BMI are not at increased risk of detrimental postoperative outcomes, such as mortality^31,32,33^ or infection,^34,35^ while others suggest the opposite.^36,37,38,39^ In our study, an increase in BMI was associated with an increased risk of *S aureus* SSIs and BSIs. This finding is in accordance with the hypothesis that suboptimal tissue oxygenation and wound hypoxia, in combination with altered antimicrobial pharmacokinetics and increased hepatic clearance of antimicrobials, which are found in obesity, increase the susceptibility of patients with higher BMI to develop postoperative infections.^37^

Another interesting finding was that mastectomy and neurosurgery, compared with orthopedic procedures, were associated with an increased risk of *S aureus* SSIs and BSIs. Although existing data show that certain surgical procedures have a higher SSI rate than other procedures,^40^ this is the first study, to our knowledge, that has compared head-to-head the risk of *S aureus* SSIs and BSIs of different surgical procedures from different surgical subspecialties in such a large cohort. It is important that health care interventions aimed at *S aureus* SSI and BSI prevention target the patient populations who have an increased risk of this outcome first. These data could help in the prioritization of these efforts.

Unexpectedly, preoperative topical *S aureus* decolonization was not associated with *S aureus* SSIs and BSIs in any of our analyses, although there is convincing evidence supporting a protective effect of preoperative *S aureus* decolonization on the occurrence of *S aureus* SSI.^41^ However, the participating sites used different preoperative decolonization strategies for different surgical procedures (universal decolonization for certain procedures regardless of colonization status, targeted decolonization in case of *S aureus* colonization or MRSA carriage only, or no decolonization), using different types of decolonization agents. Therefore, the use of topical *S aureus* decolonization treatment was probably too diverse; thus, we cannot draw any conclusions regarding the true effectiveness of preoperative *S aureus* colonization against *S aureus* SSIs and BSIs based on this finding.

Last, we also found that having nonremovable artificial body implants before surgery increased the risk of developing *S aureus* SSIs and BSIs. This finding was surprising because there is currently no published evidence to support a biological mechanism or epidemiologic link. It could be that the foreign material predisposes patients to postoperative infections by facilitating hematogenous seeding of the pathogen,^42,43^ although we do not have information to support this claim.

### Limitations

This study had some limitations that merit consideration. First, for the sample size that we included, we expected a higher incidence of *S aureus* SSIs and BSIs than we observed. Because of this, we were restricted in the number of potential etiologic factors that we could analyze. Despite this, we were still able to address important etiologic factors in our analyses, although we cannot exclude that there still might be some residual confounding present. Second, the evaluation of preoperative *S aureus* carriage was based only on colonial morphologic findings, and no additional microbiological confirmation was requested. For that reason, we cannot exclude misclassification of *S aureus* carrier status for some patients. Third, patient recruitment in certain countries and for certain surgical procedures progressed slower than we expected, which resulted in overrepresentation of patients from certain countries and surgical procedures in the study (eTables 1 and 2 in Supplement 1). This overrepresentation may have affected the generalizability of our results.

## Conclusions

The findings of this cohort study indicate that preoperative *S aureus* carriage is still a major predisposing factor for postoperative *S aureus* infection, increasing the daily risk more than 4-fold. Additionally, surgery type, an increasing BMI, and having any artificial body implants before surgery may also have an impact on the risk of developing these infections. We provide updated data that can be used to design future clinical trials of strategies aimed at *S aureus* SSI and BSI prevention or that can provide guidance on which surgical patient groups to prioritize when implementing a strategy aimed at *S aureus* SSI and BSI prevention.

## Notes

Supplement 1. **eMethods.** Supplemental methods **eTable 1.** Number of study participants per country and European sub-region **eTable 2.** Baseline characteristics of the weighted study population and original source population **eTable 3.** Weighted cumulative incidence of SA SSI/BSI within 90 days post-surgery by surgery type **eTable 4.** Unweighted cumulative incidence of SA SSI/BSI by preoperative SA colonization status **eTable 5.** Methicillin-susceptibility of colonizing strains **eTable 6.** ST types of the isolates from the sub-cohort (N = 346) **eTable 7.** Unweighted risk factor analysis for SA SSI/BSI **eTable 8.** Weighted risk factor analysis for SA SSI/BSI (keeping preoperative SA decolonization in multivariable model) **eTable 9.** Weighted Fine and Gray model for SA SSI/BSI **eTable 10.** Weighted risk factor analysis for SA SSI/BSI (complete case analysis) **eFigure 1.** Subject selection and number of isolates for the MLST analysis **eFigure 2.** Cumulative incidence function for SA SSI/BSI (unweighted data)
eReferences

Supplement 2.
Data Sharing Statement
