# Error in Author Affiliations

**DOI:** 10.1001/jamanetworkopen.2024.4564

**Published:** 2024-03-05

In the Original Investigation titled “Postoperative *Staphylococcus aureus* Infections in Patients With and Without Preoperative Colonization,”^1^ published October 31, 2023, the affiliation for author Adrian Todor has been corrected from Emergency County Hospital, Cluj-Napoca, Romania, to “Iuliu Hatieganu” University of Medicine and Pharmacy, Cluj-Napoca, Romania. This article has been corrected.^1^
