# Supplementary material for: Postoperative Staphylococcus aureus Infections in Patients With and Without Preoperative Colonization
Source: JAMA Netw Open. 2023 Oct 31;6(10):e2339793. doi: 10.1001/jamanetworkopen.2023.39793 (PMC10618839; doi:10.1001/jamanetworkopen.2023.39793)
Supplement: Supplement 1. — eMethods. Supplemental methods eTable 1. Number of study participants per country and European sub-region eTable 2. Baseline characteristics of the weighted study population and original source population eTable 3. Weighted cumulative incidence of SA SSI/BSI within 90 days post-surgery by surgery type eTable 4. Unweighted cumulative incidence of SA SSI/BSI by preoperative SA colonization status eTable 5. Methicillin-susceptibility of colonizing strains eTable 6. ST types of the isolates from the sub-cohort (N = 346) eTable 7. Unweighted risk factor analysis for SA SSI/BSI eTable 8. Weighted risk factor analysis for SA SSI/BSI (keeping preoperative SA decolonization in multivariable model) eTable 9. Weighted Fine and Gray model for SA SSI/BSI eTable 10. Weighted risk factor analysis for SA SSI/BSI (complete case analysis) eFigure 1. Subject selection and number of isolates for the MLST analysis eFigure 2. Cumulative incidence function for SA SSI/BSI (unweighted data) eReferences [file jamanetwopen-e2339793-s001.pdf]

Supplementary Online Content

Troeman DPR, Hazard D, Timbermont L, et al. Postoperative *Staphylococcus aureus* infections in patients with and without preoperative colonization. *JAMA Netw Open*. 2023;6(10):e2339793. doi:10.1001/jamanetworkopen.2023.39793

eMethods. Supplemental Methods
eTable 1. Number of study participants per country and European sub-region
eTable 2. Baseline characteristics of the weighted study population and original source population
eTable 3. Weighted cumulative incidence of SA SSI/BSI within 90 days post-surgery by surgery type
eTable 4. Unweighted cumulative incidence of SA SSI/BSI by preoperative SA colonization status
eTable 5. Methicillin-susceptibility of colonizing strains
eTable 6. ST types of the isolates from the sub-cohort (N=346)
eTable 7. Unweighted risk factor analysis for SA SSI/BSI
eTable 8. Weighted risk factor analysis for SA SSI/BSI (keeping preoperative SA decolonization in multivariable model)
eTable 9. Weighted Fine and Gray model for SA SSI/BSI
eTable 10. Weighted risk factor analysis for SA SSI/BSI (complete case analysis)
eFigure 1. Subject selection and number of isolates for the MLST analysis
eFigure 2. Cumulative incidence function for SA SSI/BSI (unweighted data)
eReferences

This supplementary material has been provided by the authors to give readers additional information about their work.

© 2023 Troeman DPR et al. *JAMA Network Open.*

eMethods. Supplemental Methods

Description of collected data

Based on the literature and clinical reasoning, we collected the following variables as they were considered potential etiological factors for *S. aureus* SSI or postoperative BSI (SA SSI/BSI) or the competing event death without SA SSI/BSI: current preoperative SA colonization status; past history of SA colonization or infection; sex; body weight and height (to calculate the body mass index [BMI] as the weight in kilograms divided by height in meters squared); age; presence of comorbidities (to calculate the Charlson comorbidity index (CCI);[1] the American Society of Anesthesiologist's (ASA) physical status classification score;[2] presence of any non-removeable implant in the body prior to surgery; receipt of preoperative decolonization treatment; receipt of immunosuppressive medication within two weeks prior to surgery; presence of surgical drain in the body after surgery; and type of surgery. None of these potential etiological factors were considered to be intermediates in the occurrence relation of other factors with the outcome SA SSI/BSI, though they could have been confounders of those occurrence relations. We also collected microbiological data, mortality data and data on the development of SSI and other infections.

Definitions:

Immunosuppressive medication was defined as any immunosuppressive/ antineoplastic/ chemotherapy medication or systemic corticosteroids, given in a dose equivalent to ≥2 weeks of daily prednisolone 20mg once daily. Sites were inquired about the decolonization strategies they employ, and the study definition was based on this information. Preoperative decolonization treatment was defined as any medication given to the patient preoperatively for the indication preoperative decolonization (as reported by the participating site), or the receipt of any (or combination) of the following medication prior to surgery (start date of decolonization should have been equal to or preceded the date of surgery): nasal mupirocin, fusidic acid (was used by some sites as an equivalent for mupirocin, and the indication for use should not have been treatment of an infection), naseptin, octenisan, octenidine, and/or chlorhexidine.

Description of the weighting methods

As reported in the manuscript, *S. aureus* carriers and non-carriers were enrolled into the study cohort in a 2:1 ratio. However, this ratio was approximately 1:3 in the overarching source population. Because the source population was a random sample of the general population, it was the population that we wanted to make inference on. For this reason we aimed to recreate the source population by weighting the study cohort subjects with the inverse probability of their inclusion in the study cohort.[3] After conducting multiple imputation for missing values in the source population (correctly screened), we fitted a logistic regression model using predictor variables available for the source population, to estimate the probability of inclusion. After this, we took the inverse of this probability as the weight. The predictor variables are listed in the table below, as well as their distributions in the source population, study cohort, and weighted study population. There is good agreement between the source and weighted population. The calculated weights were used for the incidence calculations and in the risk factor analyses in the accompanying manuscript.

Characteristic	Level	Source population (correctly screened)	Study cohort	Weighted population
N		10570	5004	9657
SA colonization (%)	Yes<br>No	3725 (35.2)<br>6845 (64.8)	3369 (67.3)<br>1635 (32.7)	3369 (34.9)<br>6288 (65.1)
Region (%)	North	2171 (20.5)	912 (18.2)	2327 (24.1)
	East	1232 (11.7)	502 (10.0)	1160 (12)
	South	4679 (44.3)	2371 (47.4)	4180 (43.3)
	West	2488 (23.5)	1219 (24.4)	1990 (20.6)
Year of inclusion (%)	2016	3 (0.0)	1 (0.0)	3 (0.0)
	2017	1173 (11.1)	505 (10.1)	858 (8.9)
	2018	5537 (52.4)	2743 (54.8)	4952 (51.3)
	2019	3857 (36.5)	1755 (35.1)	3844 (39.8)
Age (median [IQR])		66 [57;73]	66 [56; 72]	66 [56;73]

Sex (%)	Female	5401 (51.1)	2510 (50.2)	5113 (52.9)
	Male	5166 (48.9)	2494 (49.8)	4544 (47.1)
	Missing	3 (0.0)	0	0
BMI (median[IQR])		27.8 [24.8;31.5]	27.8 [24.8; 31.3]	27.8[24.9;31.3]
ASA score (%)	1	926 (8.8)	462 (9.2)	955 (9.9)
	2	4331 (41)	2152 (43)	4318 (44.7)
	3	4093 (38.7)	1909 (38.1)	3400 (35.2)
	≥4	553 (5.2)	280 (5.6)	500 (5.2)
	Missing	667 (6.3)	201 (4.0)	484 (5)
Non-removable implant present prior to surgery (%)	Yes	2549 (24.1)	1207 (24.1)	2398 (24.8)
	No	8003 (75.7)	3790 (75.8)	7242 (75.0)
	Missing	18 (0.2)	7 (0.1)	17 (0.2)
Diabetes mellitus (%)	Yes	1971 (18.6)	974 (19.5)	1739 (18)
	No	8588 (81.3)	4030 (80.5)	7918 (82)
	Missing	11 (0.1)	0	0
Type of surgery	Open heart surgery	2316 (21.9)	969 (19.4)	1772 (18.3)
	Implantable cardioverter defibrillator implantation	175 (1.7)	82 (1.6)	219 (2.3)
	Knee prosthesis surgery	1919 (18.2)	983 (19.6)	1895 (19.6)
	Hip prosthesis surgery	1669 (15.8)	857 (17.1)	1523 (15.8)
	Laminectomy	953 (9.0)	458 (9.2)	925 (9.6)
	Spinal fusion surgery	371 (3.5)	151 (3.0)	446 (4.6)
	Peripheral artery bypass surgery	618 (5.8)	296 (5.9)	438 (4.5)
	Central artery reconstruction surgery	255 (2.4)	143 (2.9)	189 (2.0)
	Mastectomy	1079 (10.2)	469 (9.4)	1031 (10.7)
	Craniotomy	534 (5.1)	290 (5.8)	519 (5.4)
	Emergency surgery	681 (6.4)	306 (6.1)	700 (7.2)
Serum sample collected (%)	Yes	9847 (93.2)	5004 (100)	9657 (100)
	No	723 (6.8)	0	0
Planned surgery occurred	Yes	10180 (96.3)	5004 (100)	9657 (100)
	No	390 (3.7)	0	0

Abbreviations: ASA. American Society of Anesthesiologist's; BMI, body mass, index; IQR, interquartile range; SA, *S. aureus*.

© 2023 Troeman DPR et al. *JAMA Network Open.*

eTable 1. Number of study participants per country and European sub-region

European sub-region	Country	Number of participants
East	Czech Republic	373
	Romania	129
North	Estonia	177
	United Kingdom	735
South	Italy	424
	Serbia	1166
	Spain	781
West	Belgium	57
	France	348
	The Netherlands	814

© 2023 Troeman DPR et al. *JAMA Network Open.*

eTable 2. Baseline characteristics of the weighted study population and original source population

	Weighted population			Source population (correctly screened)		
Characteristic	SA carrier (N=3369)	Non-carrier (N=6288)	Overall (N=9657)	SA carrier (N=3725)	Non-carriers (N=6845)	Overall (N=10570)
Sex (%)						
Female	1642 (48.7)	3471 (55.2)		1826 (49)	3575 (52.2)	5401 (51.1)
Male	1727 (51.3)	2817 (44.8)		1899 (51)	3267 (47.7)	5166 (48.9)
Missing	0	0	0	0	3 (0)	3 (0)
Implant before surgery (%)						
No	2544 (75.5)	4698 (74.7)	7242 (75.0)	2805 (75.3)	5198 (75.9)	8003 (75.7)
Yes	820 (24.3)	1578 (25.1)	2398 (24.8)	915 (24.6)	1634 (23.9)	2549 (24.1)
Missing	5 (0.1)	12 (0.2)	17 (0.2%)	5 (0.1)	13 (0.2)	18 (0.2)
Type of surgery (%)						
Cardiovascular surgery [a]	1002 (29.7)	1616 (25.7)	2618 (27.1)	1075 (28.9)	2289 (33.4)	3364 (31.8)
Orthopedic surgery [b]	1231 (36.5)	2187 (34.8)	3418 (35.4)	1383 (37.1)	2205 (32.2)	3588 (33.9)
Neurosurgery [c]	612 (18.2)	1278 (20.3)	1890 (19.6)	686 (18.4)	1172 (17.1)	1858 (17.6)
Emergency surgery	207 (6.1)	493 (7.8)	700 (7.2)	240 (6.4)	441 (6.4)	681 (6.4)
Mastectomy	317 (9.4)	714 (11.4)	1031 (10.7)	341 (9.2)	738 (10.8)	1079 (10.2)
ASA score						
1	326 (9.7)	629 (10)	955 (9.9)	370 (9.9)	556 (8.1)	926 (8.8)
2	1445 (42.9)	2873 (45.7)	4318 (44.7)	1597 (42.9)	2734 (39.9)	4331 (41)
3	1269 (37.7)	2131 (33.9)	3400 (35.2)	1370 (36.8)	2723 (39.8)	4093 (38.7)
≥4	187 (5.6)	313 (5.0)	500 (5.2)	195 (5.2)	358 (5.2)	553 (5.2)
Missing	142 (4.2)	342 (5.4)	484 (5)	193 (5.2)	474 (6.9)	667 (6.3)
Immunosuppressive medication within 2 weeks prior to surgery? (%)						
No	3194 (94.8)	6049 (96.2)	9243 (95.7)	-	-	-
Yes	171 (5.1)	235 (3.7)	406 (4.2)	-	-	-
Missing	4 (0.1)	4 (0.1)	8 (0.1)	-	-	-
History of SA colonization or infection? (%)						
No	3033 (90)	6152 (97.8)	9185 (95.1)	3343 (89.7)	6729 (98.4)	10072 (95.3)
Yes	329 (9.8)	118 (1.9)	447 (4.6)	375 (10.1)	108 (1.6)	483 (4.6)
Missing	7 (0.2)	18 (0.3)	25 (0.3)	7 (0.2)	8 (0.1)	15 (0.1)

© 2023 Troeman DPR et al. *JAMA Network Open.*

eTable 2. Baseline characteristics of the weighted study population and original source population

	Weighted population			Source population (correctly screened)		
Characteristic	SA carrier (N=3369)	Non-carrier (N=6288)	Overall (N=9657)	SA carrier (N=3725)	Non-carriers (N=6845)	Overall (N=10570)
Receipt of preoperative decolonization treatment (%)						
No	2538 (75.3)	5251 (83.5)	7789 (80.7)	-	-	-
Yes	831 (24.7)	1037 (16.5)	1868 (19.3)	-	-	-
Age (median [IQR])	65 [55;72]	67 [57;73]	66 [56;73]	65 [55;72]	67 [58;74]	66 [57;73]
Missing (%)	-	-	-	0 (0)	4 (0.1)	4 (0)
BMI (median [IQR])	27.9 [24.9; 31.6]	27.8 [24.8;31.2]	27.8[24.9;31.3]	28 [25;31.6]	27.8 [24.8;31.3]	27.8 [24.8;31.5]
Missing (%)	44 (1.3)	128	172 (1.8)	53 (1)	102 (1.5)	155 (1.5)
CCI (median [[IQR])	1[0;2]	1[0;2]	1[0;2]	-	-	-
Missing (%)	1 (0.0)	14 (0.2)	15 (0.2)	-	-	-

[a] Included: open cardiac surgery, implantable cardioverter defibrillator (ICD) implantation, peripheral artery bypass surgery, and central artery reconstructive surgery.

[b] Included: hip prosthesis and knee prosthesis surgery.

[c] Included: craniotomy, laminectomy, and spinal fusion surgery.

The variables *immunosuppressive medication; preoperative decolonization treatment,* and *CCI* were not collected in the original source population.

Abbreviations: ASA, American Society of Anesthesiologist's; BMI, body mass index; CCI, Charlson comorbidity index; IQR, interquartile range; SA, *S. aureus*.

© 2023 Troeman DPR et al. *JAMA Network Open.*

eTable 3. Weighted cumulative incidence of SA SSI/BSI within 90 days post-surgery by surgery type

Type of surgery	No. of subjects	No. of SA SSI/BSI events	Cumulative incidence per 100 patients (95% CI)	Median time (IQR) to event, d
Cardiovascular surgery	2618	40	1.53 (1.07; 2.02)	22 (13-33)
Orthopedic surgery	3418	23	0.67 (0.41; 0.97)	22 (15-31)
Neurosurgery	1890	25	1.32 (0.85; 1.85)	18 (11-26)
Emergency surgery	700	9	1.29 (0.57; 2.14)	34 (26-34)
Mastectomy	1031	22	2.13 (1.36; 3.01)	12 (10-18)

The cumulative incidences were bootstrapped. 10,000 bootstrap samples of the study cohort were made, after which the bootstrap samples were inflated using the weights. In each weighted bootstrap sample, the cumulative incidence of SA SSI/BSI for each surgery type was calculated. Using the sequence of 10,000 cumulative incidences for each surgery type, the median cumulative incidence with 95% CI (2.5th and 97.5th percentile) was derived.

Abbreviations: BSI, bloodstream infection; CI, confidence interval; IQR, interquartile range; No., number; SA, *S. aureus*; SSI, surgical site infection.

© 2023 Troeman DPR et al. *JAMA Network Open.*

eTable 4. Unweighted cumulative incidence of SA SSI/BSI by preoperative SA colonization status

***S. aureus* colonization status**	**No. of subjects**	**No. of SA SSI/BSI events**	**Cumulative incidence per 100 patients (95% CI)**	**Median time (IQR) to event, d**
SA carrier (any body location)	3369	86	2.55 (2.05; 3.09)	19 (13-33)
Non-carriers	1635	14	0.86 (0.22; 0.92)	22 (13-32.5)

The cumulative incidences were bootstrapped. 10,000 bootstrap samples of the study cohort were made. In each bootstrap sample, the cumulative incidence for SA carriers and non-carriers was calculated. The sequence of 10,000 cumulative incidences for SA carriers and non-carriers was used to derive the median cumulative incidence with 95% CI (2.5th and 97.5th percentile).

Abbreviations: BSI, bloodstream infection; CI, confidence interval; IQR, interquartile range; No., number*;* SSI, surgical site infection.

© 2023 Troeman DPR et al. *JAMA Network Open.*

eTable 5. Methicillin-susceptibility of colonizing strains

**Result**	**No. of subjects (%) (N=3138)[a]**
Colonized with MSSA only	3011 (96%)
Colonized with MSSA and MRSA	22 (0.7%)
Colonized with MRSA only	105 (3.3%)

[a] There were 231 carriers who did not have screening isolates that could be analyzed. These patients are not included in the calculation.

Abbreviations: MSSA, methicillin-susceptible *S. aureus*; MRSA: methicillin-resistant *S. aureus*; No, number.

© 2023 Troeman DPR et al. *JAMA Network Open.*

eTable 6. ST types of the isolates from the sub-cohort (N=346)

MLST type	Colonizing SA isolates for the randomly selected cohorts patients; 172 unique STs from 162 subjects (%)	Colonizing SA isolates from the patients who developed a SA SSI/BSI; 57 unique STs from 54 subjects(%)	Infecting SA isolates from the patients who developed a SA SSI/BSI, 66 unique STs from 60 subjects (%)
ST30	22 (13%)	7 (12%)	8 (12%)
ST45	20 (12%)	6 (11%)	5 (7.6%)
ST5	14 (8.1%)	3 (5.3%)	5 (7.6%)
ST8	14 (8.1%)	3 (5.3%)	8 (12%)
ST398	14 (8.1%)	7 (12%)	10 (15%)
ST15	11 (6.4%)	3 (5.3%)	1(1.5%)
ST7	8 (4.7%)	3 (5.3%)	4 (6.1%)
ST22	7 (4.1%)	3 (5.3%)	3 (4.5%)
ST582	7 (4.1%)	3 (5.3%)	2 (3.0%)
ST6	7 (4.1%)	1 (1.8%)	2 (3.0%)
ST72	4 (2.3%)	3 (5.3%)	2 (3.0%)
ST34	3 (1.7%)	2 (3.5%)	1 (1.5%)
ST10	4 (2.3%)	0	0
ST121	4 (2.3%)	0	0
ST12	1 (0.6%)	1 (1.8%)	1 (1.5%)
ST4302	0	1 (1.8%)	1 (1.5%)
Other ST types	32 (19%)	11 (19%)	13 (20%)

Subjects with multiple isolates (colonizing or infecting) were included more than once if they had at least 2 isolates with different STs (then they were counted in twice in the table above). Subjects with multiple isolates of the same ST were only counted in once.

Abbreviations: ST, sequence type.

© 2023 Troeman DPR et al. *JAMA Network Open.*

eTable 7. Unweighted risk factor analysis for SA SSI/BSI

	**Univariable analysis for the association with SA SSI/BSI**		**Univariable analysis for the association with death without SA SSI/BSI**		**Multivariable analysis for the association with SA SSI/BSI[e]**	
Risk factor	Crude HR (95% CI)	P-value	Crude HR (95% CI)	P-value	Adjusted HR (95% CI)	P-value
Preoperative SA colonization status [a]						
Colonized	3.01 (1.70-5.34)	**<0.001**	1.07 (0.68-1.70)	0.78	2.84 (1.60-5.04)	**<0.001**
Non-colonized	1 [Reference]		1 [Reference]		1 [Reference]	
Sex						
Male	1.17 (0.78-1.75)	0.45	1.47 (0.95-2.28)	**0.08**	1.30 (0.80-2.11)	0.29
Female	1 [Reference]		1 [Reference]			
Non-removable implant prior to surgery	1.48 (0.96-2.27)	**0.08**	0.58 (0.31-1.08)	**0.08**	1.87 (1.18-2.95)	**0.008**
Type of surgery						
Cardiovascular surgery	2.52 (1.39-4.56)	**0.003**	5.37 (2.61-11.04)	**<0.001**	2.73 (1.33-5.58)	**0.007**
Mastectomy	4.71 (2.32-9.59)	**<0.001**	0.68 (0.08-5.54)	0.71	5.99 (2.66-13.46)	**<0.001**
Neurosurgery	2.13 (1.04-4.36)	**0.04**	14.35 (6.51-31.61)	**<0.001**	2.28 (1.06-4.94)	**0.04**
Emergency surgery	2.10 (0.82-5.42)	**0.12**	6.86 (2.99-15.73)	**<0.001**	2.81 (1.05-7.48)	**0.04**
Orthopedic surgery	1 [Reference]	-	1 [Reference]	-	1 [Reference]	-
Having no postoperative drains	0.75 (0.47-1.18)	0.21	1.67 (1.04-2.67)	**0.03**	0.96 (0.58-1.56)	0.85
ASA status						
ASA 1	0.69 (0.30-1.58)	0.38	0.95 (0.21-4.43)	0.95	0.63 (0.27-1.49)	0.29
ASA 3	0.99 (0.63-1.57)	0.97	3.44 (1.86-6.36)	**<0.001**	0.75 (0.43-1.32)	0.32
ASA ≥ 4	1.39 (0.61-3.17)	0.44	14.05 (6.91-28.60)	**<0.001**	0.97 (0.38-2.48)	0.95
ASA 2	1 [Reference]		1 [Reference]			
Immunosuppressive medication within 2 weeks of surgery?	1.39 (0.63-3.03)	0.41	4.91 (2.93-8.22)	**<0.001**	1.58 (0.69-3.63)	0.27
Prior history of SA colonization or infection	1.02 (0.45-2.29)	0.97	1.89 (0.53-6.73)	0.32	Not included	Not applicable
Preoperative decolonization	1.39 (0.82-2.36)	0.22	1.30 (0.56- 3.01)	0.53	Not included	Not applicable
Age[b]	0.99 (0.98-1.01)	0.19	1.06 (1.04- 1.08)	**<0.001**	0.99 (0.97-1.01)	0.28
BMI[c]	1.04 (1.01-1.08)	**0.02**	0.95 (0.91-1.00)	**0.03**	1.05 (1.02-1.09)	**0.006**
CCI[d]	1.16 (1.04-1.30)	**0.007**	1.53 (1.41- 1.67)	**<0.001**	1.12 (0.99-1.28)	**0.08**

[a] Status prior to surgery based on screening of the nose, throat and perineum;
[b] per 1-year increase in age [range 18-99];
[c] per 1-point increase in BMI [range 13.5-65.8];
[d] per 1-point increase in the CCI [range 0-12].
[e] Risk factors with a p-value ≤ 0.157 in either univariable analyses, were selected for the multivariable analysis.
Abbreviations: ASA, American Society of Anesthesiologist's; BMI, body mass index; BSI, bloodstream infection; CCI, Charlson Comorbidity Index; CI, confidence interval; HR, hazard ratio; SA, *S. aureus*; SSI, Surgical site infection.

© 2023 Troeman DPR et al. *JAMA Network Open.*

eTable 8. Weighted risk factor analysis for SA SSI/BSI (keeping preoperative SA decolonization in multivariable model)

	Univariable analysis for the association with SA SSI/BSI		Univariable analysis for the association with death without SA SSI/BSI		Multivariable analysis for the association with SA SSI/BSI[e]	
Risk factor	Crude HR (95% CI)	P-value	Crude HR (95% CI)	P-value	Adjusted HR (95% CI)	P-value
Preoperative SA colonization status [a]						
Colonized	4.56 (2.26-9.21)	**<0.001**	1.17 (0.70-1.97)	0.55	4.39 (2.21-8.73)	**<0.001**
Non-colonized	1 [Reference]		1 [Reference]		1 [Reference]	
Sex						
Male	1.35 (0.79-2.30)	0.27	1.41 (0.83-2.39)	0.20	Not included	Not applicable
Female	1 [Reference]		1 [Reference]			
Non-removable implant prior to surgery	1.56 (0.91-2.67)	**0.10**	0.48 (0.24-0.99)	**0.05**	2.00 (1.15-3.50)	**0.01**
Type of surgery						
Cardiovascular surgery	2.13 (1.10-4.12)	**0.03**	5.55 (2.34-13.21)	**<0.001**	1.91 (0.86-4.24)	0.11
Mastectomy	4.12 (1.52-11.20)	**0.006**	0.44 (0.06-3.44)	0.43	5.13 (1.87-14.13)	**0.002**
Neurosurgery	2.37 (1.01-5.55)	**0.05**	12.80 (5.23-31.33)	**<0.001**	2.48 (1.08-5.66)	**0.03**
Emergency surgery	1.78 (0.54-5.84)	0.35	7.40 (2.37-23.06)	**0.001**	2.42 (0.67-8.67)	0.18
Orthopedic surgery	1 [Reference]	-	1 [Reference]	-	1 [Reference]	-
Having no postoperative drains	0.82 (0.47-1.41)	0.47	1.52 (0.80-2.88)	0.21	Not included	Not applicable
ASA status						
ASA 1	0.73 (0.32-1.71)	0.47	0.63 (0.13-3.06)	0.57	0.58 (0.25-1.34)	0.21
ASA 3	1.46 (0.80-2.69)	0.22	3.30 (1.48-7.40)	**0.004**	1.49 (0.63-3.55)	0.37
ASA ≥ 4	1.66 (0.67-4.12)	0.28	7.91 (3.17-19.76)	**<0.01**	1.70 (0.59-4.88)	0.32
ASA 2	1 [Reference]		1 [Reference]			
Immunosuppressive medication within 2 weeks prior to surgery?	1.77 (0.72-4.36)	0.21	3.88 (2.29- 6.55)	**<0.001**	1.74 (0.62-4.92)	0.30
Prior history of SA colonization or infection	1.11 (0.46-2.67)	0.82	2.79 (0.47-16.50)	0.26	Not included	Not applicable
Preoperative decolonization	1.35 (0.75-2.44)	0.32	1.09 (0.49- 2.47)	0.83	0.96 (0.54-1.71)	0.88
Age[b]	0.99 (0.97-1.00)	**0.03**	1.03 (0.99- 1.06)	**0.12**	0.98 (0.97-1.00)	0.08
BMI[c]	1.04 (1.01-1.08)	**0.009**	0.97 (0.90-1.05)	0.48	1.05 (1.01-1.08)	**0.008**
CCI[d]	1.16 (1.05-1.28)	**0.003**	1.49 (1.36- 1.64)	**<0.001**	1.09 (0.97-1.22)	0.17

[a] Colonization status prior to surgery based on SA screening of the nose, throat and perineum;
[b] per 1-year increase in age [range 18-99];
[c] per 1-point increase in BMI [range 13.5-65.8];
[d] per 1-point increase in the CCI [range 0-12].
[e] Risk factors with a p-value ≤ 0.157 in either univariable analyses, were selected for the multivariable analysis.
Abbreviations: ASA, American Society of Anesthesiologist's; BMI, body mass index; BSI, bloodstream infection; CCI, Charlson Comorbidity Index; CI, confidence interval; HR, hazard ratio; SA, *S. aureus*; SSI, Surgical site infection.

© 2023 Troeman DPR et al. *JAMA Network Open.*

eTable 9. Weighted Fine and Gray model for SA SSI/BSI

	**Univariable analysis for the association SA SSI/BSI**		**Multivariable analysis for the association with SA SSI/BSI**	
Risk factor	Crude SDHR (95% CI)	P-value	Adjusted SDHR (95% CI)	P-value
Preoperative SA colonization status [a]				
Colonized	4.55 (2.25-9.21)	**<0.001**	4.37 (2.18-8.76)	**<0.001**
Non-colonized	1 [Reference]		1 [Reference]	
Sex				
Male	1.35 (0.79-2.29)	0.27	Not included	Not applicable
Female	1 [Reference]			
Non-removable implant prior to surgery	1.57 (0.92-2.68)	**0.10**	2.00 (1.15-3.49)	**0.01**
Type of surgery				
Cardiovascular surgery	2.10 (1.09-4.08)	**0.03**	1.88 (0.85-4.17)	0.12
Mastectomy	4.12 (1.51-11.21)	**0.006**	5.10 (1.86-14.01)	**0.002**
Neurosurgery	2.34 (1.00-5.48)	**0.05**	2.42 (1.06-5.48)	**0.04**
Emergency surgery	1.77 (0.54-5.82)	0.35	2.41 (0.66-8.80)	0.18
Orthopedic surgery	1 [Reference]	-	1 [Reference]	-
Having no postoperative drains	0.82 (0.47-1.41)	0.47	Not included	Not applicable
ASA status				
ASA 1	0.73 (0.32-1.70)	0.47	0.58 (0.25-1.33)	0.20
ASA 3	1.46 (0.79-2.68)	0.23	1.49 (0.63- 3.56)	0.37
ASA ≥ 4	1.61 (0.65-3.98)	0.30	1.62 (0.57-4.66)	0.37
ASA 2	1 [Reference]	-	1 [Reference]	-
Immunosuppressive medication within 2 weeks of surgery?	1.75 (0.71-4.31)	0.22	1.72 (0.60-4.87)	0.31
Prior history of SA colonization or infection	1.09 (0.45-2.63)	0.84	Not included	Not applicable
Preoperative decolonization	1.35 (0.75-2.44)	0.32	Not included	Not applicable
Age[b]	0.99 (0.97-1.00)	**0.03**	0.98 (0.97-1.00)	0.07
BMI[c]	1.04 (1.01-1.08)	**0.009**	1.05 (1.01-1.08)	**0.008**
CCI[d]	1.15 (1.05-1.27)	**0.004**	1.08 (0.96-1.22)	0.19

[a] Colonization status prior to surgery based on *S. aureus* screening of the nose, throat and perineum;
[b] per 1-year increase in age [range 18-99];
[c] per 1-point increase in BMI [range 13.5-65.8];
[d] per 1-point increase in the CCI [range 0-12].
Abbreviations: ASA, American Society of Anesthesiologist’s; BMI, body mass index; BSI, bloodstream infection; CCI, Charlson Comorbidity Index; CI, confidence interval; SDHR, subdistribution hazard ratio; SSI, Surgical site infection.

© 2023 Troeman DPR et al. *JAMA Network Open.*

eTable 10. Weighted risk factor analysis for SA SSI/BSI (complete case analysis)

	**Univariable analysis for the association with SA SSI/BSI**		**Univariable analysis for the association with death without SA SSI/BSI**		**Multivariable analysis for the association with SA SSI/BSI[e]**	
Risk factor	Crude HR (95% CI)	P-value	Crude HR (95% CI)	P-value	Adjusted CSHR (95% CI)	P-value
Preoperative SA colonization status [a]						
Colonized	4.56 (2.26-9.21)	**<0.001**	1.17 (0.70-1.97)	0.55	4.04 (2.02-8.10)	**<0.001**
Non-colonized	1 [Reference]		1 [Reference]		1 [Reference]	
Sex						
Male	1.35 (0.79-2.30)	0.27	1.41 (0.83-2.39)	0.20	Not included	Not applicable
Female	1 [Reference]		1 [Reference]			
Non-removable implant prior to surgery	1.56 (0.91-2.66)	**0.11**	0.48 (0.24-0.99)	**0.05**	2.15 (1.21-3.80)	**0.008**
Type of surgery						
Cardiovascular surgery	2.13 (1.10-4.12)	**0.03**	5.55 (2.34-13.21)	**<0.001**	1.97 (0.85-4.54)	0.11
Mastectomy	4.12 (1.52-11.20)	**0.006**	0.44 (0.06-3.44)	0.43	5.77 (2.05-16.24)	**<0.001**
Neurosurgery	2.37 (1.01-5.55)	**0.05**	12.80 (5.23-31.33)	**<0.001**	3.12 (1.34-7.28)	**0.008**
Emergency surgery	1.78 (0.54-5.84)	0.35	7.40 (2.37-23.06)	**0.001**	2.61 (0.70-9.77)	0.15
Orthopedic surgery	1 [Reference]	-	1 [Reference]		1 [Reference]	
Having no postoperative drains	0.82 (0.47-1.41)	0.47	1.52 (0.80-2.88)	0.21	Not included	Not applicable
ASA status						
ASA 1	0.75 (0.32-1.75)	0.50	0.57 (0.12-2.73)	0.48	0.63 (0.28-1.43)	0.27
ASA 3	1.47 (0.78-2.77)	0.23	3.41 (1.50-7.76)	**0.004**	1.53 (0.61-3.84)	0.36
ASA ≥ 4	1.74 (0.70-4.33)	0.23	8.52 (3.33-21.77)	**<0.001**	1.90 (0.63-5.78)	0.26
ASA 2	1 [Reference]		1 [Reference]		1 [Reference]	
Immunosuppressive medication within 2 weeks of surgery?	1.77 (0.72-4.36)	0.21	3.88 (2.29-6.55)	**<0.001**	1.29 (0.38-4.35)	0.69
Prior history of SA colonization or infection	1.11 (0.46-2.67)	0.82	2.79 (0.47-16.48)	0.26	Not included	Not applicable
Preoperative decolonization	1.35 (0.75-2.44)	0.32	1.09 (0.49-2.47)	0.83	Not included	Not applicable
Age[b]	0.99 (0.97-1.00)	**0.03**	1.03 (0.99-1.06)	**0.12**	0.99 (0.97-1.01)	0.16
BMI[c]	1.04 (1.01-1.08)	**0.01**	0.97 (0.90-1.05)	0.48	1.05 (1.01-1.08)	**0.008**
CCI[d]	1.16 (1.05-1.28)	**0.003**	1.49 (1.36-1.64)	**<0.001**	1.08 (0.96-1.21)	0.22

[a] Colonization status prior to surgery based on *S. aureus* screening of the nose, throat and perineum;
[b] per 1-year increase in age [range 18-99];
[c] per 1-point increase in BMI [range 13.5-65.8];
[d] per 1-point increase in the CCI [range 0-12].
[e] Risk factors with a p-value ≤ 0.157 in either univariable analyses, were selected for the multivariable analysis.
Abbreviations: ASA, American Society of Anesthesiologist's; BMI, body mass index; BSI, bloodstream infection; CCI, Charlson Comorbidity Index; CI, confidence interval; HR, hazard ratio; SA, *S. aureus*; SSI, Surgical site infection.

© 2023 Troeman DPR et al. *JAMA Network Open.*

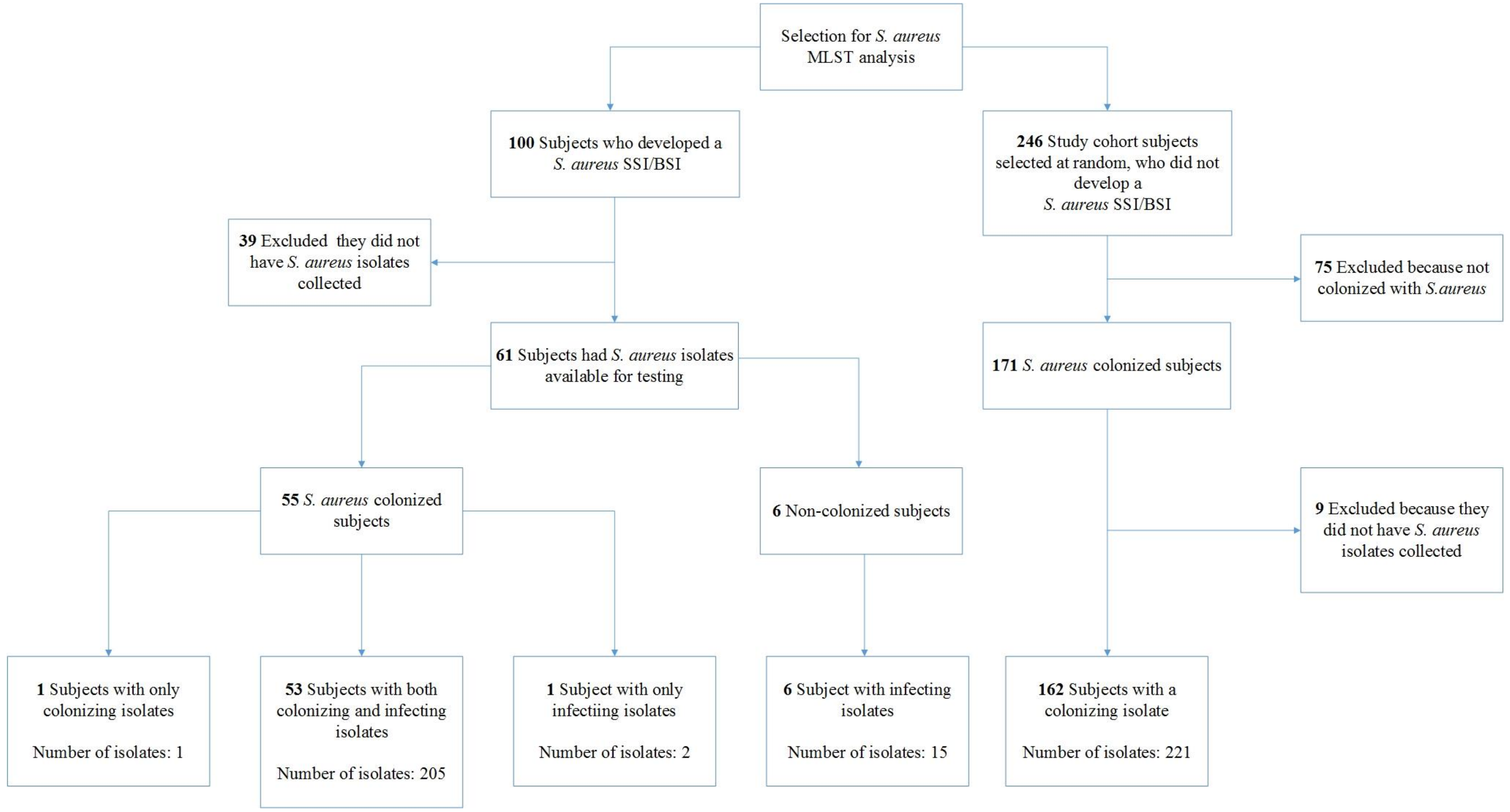

eFigure 1. Subject selection and number of isolates for the MLST analysis

© 2023 Troeman DPR et al. *JAMA Network Open.*

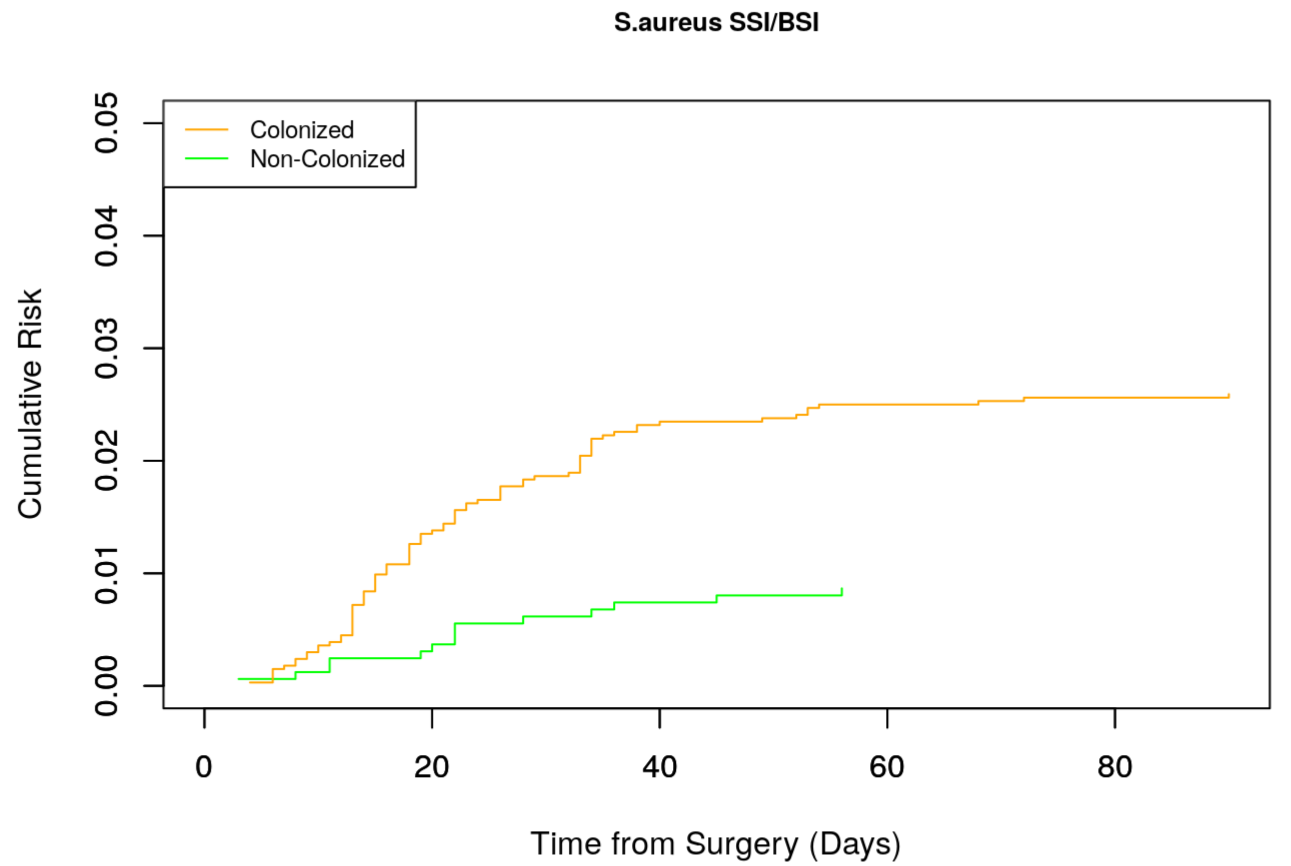

eFigure 2. Cumulative incidence function for SA SSI/BSI (unweighted data)

The follow-up time was 90 days for both SA colonized and non-colonized subjects. However, the cumulative risk for non-colonized subjects did not change after day 56.

Abbreviations: BSI, bloodstream infection; SA, S. aureus; SSI, Surgical site infection.

© 2023 Troeman DPR et al. *JAMA Network Open.*

eReferences

1. Charlson ME, Pompei P, Ales KL, MacKenzie CR. A new method of classifying prognostic comorbidity in longitudinal studies: Development and validation. *J Chronic Dis*. 1987;40(5):373-383. doi: 10.1016/0021-9681(87)90171-8.

2. Mayhew D, Mendonca V, Murthy BVS. A review of ASA physical status - historical perspectives and modern developments. *Anaesthesia*. 2019;74(3):373-379. doi: 10.1111/anae.14569.

3. Lee ES, Forthofer RR. *Analyzing complex survey data.* Vol 71. 2nd edition ed. SAGE Publications, Inc; 2005.

© 2023 Troeman DPR et al. *JAMA Network Open.*
