# Supplementary material for: Postoperative Staphylococcus aureus Infections in Patients With and Without Preoperative Colonization
Source: JAMA Netw Open. 2023 Oct 31;6(10):e2339793. doi: 10.1001/jamanetworkopen.2023.39793 (PMC10618839; doi:10.1001/jamanetworkopen.2023.39793)
Supplement: Supplement 2. — Data Sharing Statement [file jamanetwopen-e2339793-s002.pdf]

Data Sharing Statement

Troeman. Postoperative Staphylococcus aureus Infections in Patients With and Without Preoperative Colonization. *JAMA Netw Open*. Published October 31, 2023. doi:10.1001/jamanetworkopen.2023.39793

Data
Data available: No

Additional Information
Explanation for why data not available: The data sets generated and analyzed during the current study are not publicly available for confidentiality reasons but are available from the corresponding author upon scientific review and approval of the request by the Study's Scientific Committee.
